# Multiple neural tube defects in the same patient with no neurological deficit

**DOI:** 10.4103/1817-1745.66677

**Published:** 2010

**Authors:** Arun Tungaria, Arun Kumar Srivastav, Ashok K. Mahapatra, Raj Kumar

**Affiliations:** Department of Neurosurgery, Sanjay Gandhi Post Graduate Institute of Medical Sciences, Lucknow, India; 1 Department of Neurosurgery, AIIMS, Delhi, India

**Keywords:** Dermal sinus, meningomyelocele, neral tube defect, tethered cord

## Abstract

Congenital deformities involving the coverings of the nervous system are called neural tube defects (NTDs). NTD can be classified as neurulation defects, which occur by stage 12, and postneurulation defects. Cervical meningocele and myelomeningocele are rare spinal dysraphic lesions. Unlike lumbosacral dysraphic lesions, there is often no neurologic deficits and thus the subtle features of cervical cord tethering may be overlooked on imaging. The presence of meningomyelocele and/or encephaloceles at multiple (two or more) sites along the vertebral axis is a very rare event occurring in <1% of cases. Less than 10 cases have been described in the published literature. We are reporting a case of multiple NTD in same patient with no neurological deficit.

## Introduction

Congenital deformities involving the coverings of the nervous system are called neural tube defects (NTDs). NTD can be classified as neurulation defects, which occur by stage 12, and postneurulation defects.[1] Neurulation defects are characterized by the absence of a skin covering over the defect and includes craniorachischisis, anencephaly and meningomyelocele. After neurulation, the ectoderm is separate from the neural tube and defects occurring after this stage have a covering of skin. The term spinal dysraphism applies to all forms of closed and open spina bifida and does not depend on whether a skin cover is present or not. This is the most common type of NTD. Cervical meningocele and myelomeningocele are rare spinal dysraphic lesions. Unlike lumbosacral dysraphic lesions, there is often no neurologic deficit; thus, the subtle features of cervical cord tethering may be overlooked on imaging. The presence of meningomyelocele and/or encephaloceles at multiple (two or more) sites along the vertebral axis is a very rare event occurring in <1% of cases. Less than 10 cases have been described in the published literature. A series of seven cases of multiple neural tube defects (MNTDs) is the largest so far on these rare anomalies.[2] We are reporting a case of multiple NTD in the same patient with no neurological deficit.

## Case Report

The patient is a 4-year-old girl from Muradabad, UP, with a history of cystic posterior neck mass present since birth, which was 3 cm × 2 cm × 2 cm in dimension. In February 2009, she was admitted to the Department of Neurosurgery, SGPGIMS, for management of the neck mass. Her perinatal history was uneventful.

**Figure 1:**
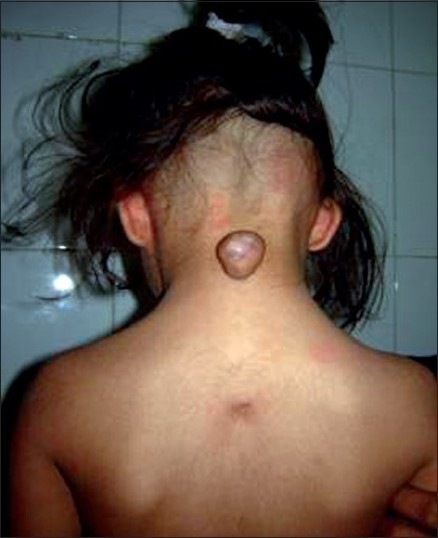
Patient having swelling in the cervical region, which was present since birth, and sinus in the thoracic region in midline

There was no history of pain in mass, no history of weakness in any limb, no history of difficulty in voiding and no history of delayed milestones. Parents had a nonconsanguineous marriage. Physical examination revealed a posterior midline rounded neck mass, nontender, skin covering of size 3 cm wide × 2 cm long × 2 cm thick [Figure 1]. It was soft to firm in consistency, nonfluctuant and nontransilluminant, without cough impulse. There was no sign of cerebrospinal fluid leakage. A dermal sinus was present at the midthoracic level.

The child had no focal neurological deficit and was playful and interested in her surroundings. Tone and bulk was normal in all four limbs and power was grade 5. Growth parameters were normal, including head circumference. Developmental assessment revealed normal developmental milestones.

**Figure 2:**
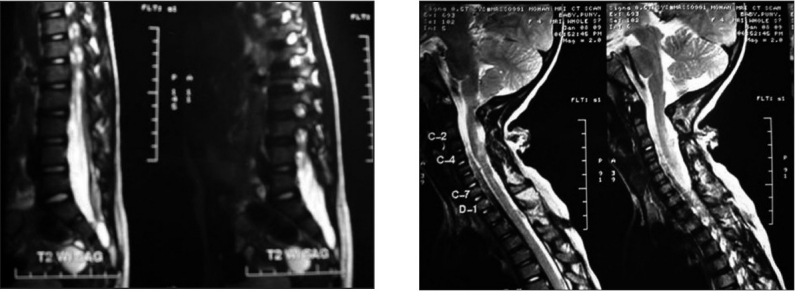
Magnetic resonance imaging (MRI) of the spine showing tethering of the cord with lipomatous filum terminale and cervicothoracic MRI showing swelling at the C2–C4 level and sinus at the D4 level

Plain cervical X-ray revealed no gross lamina or bony defects. Magnetic resonance imaging (MRI) of the cervical spine showed a heterointense lesion at the C3–C4 level extending to the subcutaneous tissue of the posterior neck compatible with a cervical meningocele [Figure 2].

A well-defined dermal sinus tract (DST) was present at the level of D3–D4 extending to the thecal sac. MRI of the lumbar spine showed thickened filum terminale with low-lying cord at L4 [Figure 2]. The spinal cord was otherwise normal with no chiari malformation and split cord malformation. Brain MRI revealed no hydrocephalus or any other anomaly.

Excision of cervical meningocele with excision of dorsal dermal sinus with lumber laminoplasty and detethering of lipomatous filum was performed under general anesthesia. The patient was positioned prone with head stabilized using a head frame. Surgery was performed in three steps in the same sitting. STEP 1: a midline vertical incision was given at the C3–C4 level and normal laminae were exposed one level above and below the swelling in the neck. Laminectomy was carried out at the same level and the meningocele sac was exposed and defined. Durotomy was performed and the sac was opened, which revealed that there was no neural tissue present in it. It was attached with cord by a thin fibrous band that was coagulated and excised. There was a small cyst present intradurally at this level looking like a neurenteric cyst, which was also excised. Hemostasis was achieved, the dura was closed and the overlying layers were closed. STEP 2: after applying an elliptical incision around the dorsal dermal sinus, it was traced and D3 and partial D4 laminectomy was performed. The sinus tract had multiple connections; one was going into the muscle plane and two of them were attached to the lamina of D4. The thickest of them was going to the cord and was attached to the cord with a thin fibrous band, which was completely cut and the wound was closed in layers. STEP 3: a 5-cm-long vertical incision was given at the lumbar spinous processes of the third to fifth vertebrae. L3–L5 laminotomy was performed and the dura was opened. There was thickened lipomatous filum terminale of size 5.0 cm × 3.0 cm. It was divided at its upper level after separating nerve roots from it. The cord was pulled upwards, the lipomatous filum was excised, hemostasis was achieved and the dura and the overlying layers were closed. Postoperative recovery was uneventful except urinary tract infections, which responded to culture-based antibiotics.

## Discussion

Multiple NTDs in the same patient has been reported very rarely in the literature. We are reporting a unique case of multiple NTDs in the same patient. Cervical meningocele, neurenteric cyst, DST at the thoracic level and lipomatous filum terminale causing tethering of the cord were present in this case. Interestingly, the patient had no neurological deficits.

Cervical meningocele and myelomeningocele are rare lesions that comprise only a small proportion of neural tube anomalies. Previous studies have reported that only 3.9–8.0% of spina bifida cystica occurred in the cervical region.[3–5]

DSTs are an uncommon form of spinal dysraphism whose cause is attributed to a failure of dysjunction during fetal development. Normally, the cutaneous ectoderm that ultimately forms the skin and dermal appendages separates from the neuroectoderm, which forms the spinal cord sometime between the third and eighth week of gestation[6–8] This process, referred to as dysjunction, allows for the insertion of the mesoderm, which then forms the vertebral column and underlying musculature that separate the skin from the spinal cord. When a focal failure of dysjunction occurs, a persistent connection, or tract, between the skin elements and the underlying neural structures is established. This tract is lined by stratified squamous epithelium encased in dermal tissue and terminating on or near the neural structures[9] The dysjunction theory supports Mount’s observation in 1949 that the dermatomal level of the defect often corresponds to the neural level of the central nervous system structures with which the tract communicates.[10]

DSTs have been reported all along the midline neuroaxis, from the nasion and occiput down to the lumbar and sacral regions. A review of all published cases of congenital spinal DSTs in 1990 reported that 1% of all tracts along the spine were cervical, 10% were thoracic, 41% were lumbar and 35% were lumbosacral.[11]

Congenital dermal sinuses are rarely located in the dorsal regi. Patients may present with meningitis and/or mass effect. Secondary to intradural lesions, this can be epidermoid, dermoid, teratoma or abscess.[12,13] In our case, DST was present at the midthoracic level and it had multiple connections.

## Conclusion

The presence of NTD at multiple sites is a very rare event. Only few cases of multiple NTDs have been described in the world literature in the form of isolated case reports. Our case is unique and is probably the first case where multiple and different types of NTD are present in the same patient, without any neurological deficit. It will be mandatory and interesting to follow this patient to see for chances of retethering at every operative site.
